# 

**Published:** 1918-03-16

**Authors:** 


### CONTENTS.

PAGE

**Alcohol and the Human Body** ... ... ... 495

**East and West in the Medical School** ... 496

**Hospital and Institutional News** ... ... ... 497

A Fine Speech—" The Crowning Act of Hindrance "—The Prudential Assurance Company—Sir Thomas Dewey—Patients' Fees or District Contributions—From the Points of the Compass—Dry-Rot in the Building—Homœopathic Hospitals and the College of Nursing—A Maternity Hospital's Good Fortune—The Vicar of Brighton's Dictum—A Rash M.P.—A Hospital Rock-Garden—Return of Secretary from the Front—Medical " Cliquism " in Dudley—Mr. Hayes Fisher on Laboratories—The *Searchlight* and the Food Problem—*Mudhook*: A Divisional Journal—This Week's Drug Market.

**Men, Women, and Bastards** ... ... ... ... 501

Some Pressing Questions of Great Importance.

**Lord Lister** ... ... ... ... ... ... ... 503

VIII. Through Pus to Surgical Cleanliness.

**His Majesty the Pig** .. ... ... ... 505

His Paramount Importance in the Great War.

PAGE

**Nursing Affairs in Ireland** ... ... ... ... ... 506

What Nurses Should Seek and Find.

**The Matrons' and Sisters' Department** ... 507

The Growth of the Nursing Profession: V. Knowledge and Practice in Nursing.

Round the Hospitals.

The New Matron of King's College Hospital—Nurses' Memorial Service at St. Paul's—College Election: Instructions to Members—Nurses as Hospital Stewards—M.A.B. Nursing: Its National Importance.

**The College of Nursing Election** ... ... ... 510

**The College of Nursing (Limited by Guarantee)** 511

List No. 5 of Training-Schools of Registered Nurses.

**The British Women's Hospital Committee** ... 512

A Short History by the Viscountess Cowdray.

**The Institution Garden** ... ... ... ... 513

I. What to Do and How to Do it.

**War Charities** ... ... ... ... ... ... ... 514

**Education in Food Economy** ... ... ... 515

**The Editor's Letter-Box** ... ... ... ... 516

